# Towards integrative physiological monitoring of the critically ill: from cardiovascular to microcirculatory and cellular function monitoring at the bedside

**DOI:** 10.1186/cc11503

**Published:** 2013-03-12

**Authors:** Abele Donati, Dick Tibboel, Can Ince

**Affiliations:** 1 Department of Intensive Care, Erasmus MC, University Medical Center Rotterdam, 's-Gravendijkwal 230, 3015 CE Rotterdam, the Netherlands; 2 Department of Biomedical Science and Public Health Anesthesia and ICU, Università Politecnica delle Marche, via Tronto 10, 60126 Torrette di Ancona, Italy; 3 Intensive Care and Department of Pediatric Surgery, Erasmus Medical Center - Sophia Children's Hospital, Postbox 2040, 3000 CA, Rotterdam, the Netherlands

## Abstract

Current hemodynamic monitoring of critically ill patients is mainly focused on monitoring of pressure-derived hemodynamic variables related to systemic circulation. Increasingly, oxygen transport pathways and indicators of the presence of tissue dysoxia are now being considered. In addition to the microcirculatory parameters related to oxygen transport to the tissues, it is becoming increasingly clear that it is also important to gather information regarding the functional activity of cellular and even subcellular structures to gain an integrative evaluation of the severity of disease and the response to therapy. Crucial to these developments is the need to provide continuous measurements of the physiological and pathophysiological state of the patient, in contrast to the intermittent sampling of biomarkers. As technological research and clinical investigations into the monitoring of critically ill patients have progressed, an increasing amount of information is being made available to the clinician at the bedside. This complexity of information requires integration of the variables being monitored, which requires mathematical models based on physiology to reduce the complexity of the information and provide the clinician with a road map to guide therapy and assess the course of recovery. In this paper, we review the state of the art of these developments and speculate on the future, in which we predict a physiological monitoring environment that is able to integrate systemic hemodynamic and oxygen-derived variables with variables that assess the peripheral circulation and microcirculation, extending this real-time monitoring to the functional activity of cells and their constituents. Such a monitoring environment will ideally relate these variables to the functional state of various organ systems because organ function represents the true endpoint for therapeutic support of the critically ill patient.

## The rise of cardiovascular monitoring

In the early 1960s, Weil realized the importance of continuous monitoring of physiological parameters coupled with calculations to provide real-time information on the hemodynamic status of patients at the bedside. One can argue that this characteristic - namely, the continuous monitoring and support of physiological variables - defines the health of critically ill patients. The introduction by Weil and Safar of monitors attached to digital computers to continuously monitor respiratory and hemodynamic measurements was a defining moment in the development of critical care medicine [1]. This technology, used in conjunction with the pulmonary artery catheter introduced by Swan [2], provided the intensivist with a powerful platform to semicontinuously monitor the functional state of the heart as the main motor driving systemic circulation. By including measurements of arterial and mixed venous gas analysis, the arterial oxygen content and the mixed venous oxygen content could easily be calculated. Consequently, oxygen delivery and oxygen consumption could be calculated from known formulae, and a target for titration therapy was formulated.

Shoemaker was the main proponent of driving systemic circulation by targeting high values of cardiac output, oxygen delivery and oxygen consumption. The basic idea behind this approach was that maximizing the oxygen delivery of the systemic circulation would ensure ample oxygen for the organ beds at risk. In initial studies, Shoemaker utilized the hemodynamic data obtained from the pulmonary artery catheter in high-risk adult surgical patients before, during and after surgical procedures. From these observational data, he established a protocol using supernormal values for cardiac output, oxygen delivery and oxygen consumption as the therapeutic goals. Indeed, this approach seemed to be favorable in surgical patients because it resulted in improved outcomes [3]. Donati and colleagues demonstrated that this approach was also successful in reducing morbidity and the length of hospital stay in high-risk surgical patients [4]. However, the effectiveness of this strategy in other critically ill patients remains controversial. Gattinoni and colleagues, for example, found no difference between patients who were treated with a protocol that targeted normal values for cardiac output, oxygen delivery and oxygen consumption, supernormal values or mixed venous saturation >70% [5]. Hayes and colleagues found an increased mortality in patients who were treated using the supranormal values protocol [6]. The condition of normal or reduced oxygen extraction seemed to be a key hemodynamic component that contraindicated targeting supranormal values of oxygen delivery. Vincent suggested that the application of a dobutamine challenge to identify the effect of increasing systemic oxygen delivery was an effective strategy to achieve hemodynamic optimization for the critically ill patient [7].

## Adding the peripheral circulation to the equation

Simply targeting the parameters related to the systemic circulation was ineffective in resuscitating the various organ systems because the microvasculature was unable to effectively regulate the flow of oxygen-carrying blood to match regional needs. This inability is presumably caused by the pathogenic action of the inflammatory mediators, reactive oxygen species and hypoxemia on vascular regulatory mechanisms, such as autoregulation. This dysfunction is augmented by certain therapies such as intravascular fluids and vasoactive mediators that override the endogenous physiological mechanisms regulating homeostasis. The consequent mismatch has its impact not only between different organ systems but also at the level of the microcirculation. This defect manifests itself as a reduced oxygen extraction deficit that is characterized by shunting within vulnerable, weak micro-circulatory units and organ beds. Reduced extraction results in elevated venous oxygen levels in the presence of signs of regional dysoxia, such as elevated levels of lactate and elevated tissue carbon dioxide (CO_2_) [8].

Weil, in advance of experimental evidence, first understood the origin of the sequence of events relating to oxygen transport dysfunction during circulatory failure [9,10]. He classified four states of shock: hypovolemic, obstructive, cardiogenic and distributive. All of these states indicate that the ultimate target of shock is the cellular starvation of oxygen availability. The first three states of shock, however, are associated with the reduction in cardiac output that is the primary cause of the ensuing tissue dysoxia. Distributive shock can occur in the presence of a normal or even elevated cardiac output and describes a defect in the vascular trafficking of cardiac output between and within the various organ beds, resulting in local tissue dysoxia in the presence of otherwise normal systemic hemodynamics. When such a distributive defect occurs, increased cardiac output is ineffective in resolving the regional tissue dysoxia. In their editorial 'Expanding from the Macro to the Microcirculation', Weil and Tang identified the need to monitor the microcirculation to monitor and treat distributive shock [11]. This realization, in combination with the idea that microcirculatory dysfunction leads to organ dysfunction, formed the basis for the appreciation of the microcirculation as a central focus in the pathogenesis of multiorgan dysfunction [12].

The need to monitor hemodynamic and oxygen-derived variables of the peripheral circulation was demonstrated by the introduction of CO_2 _gastric tonometry by Fiddian-Green and Baker to identify splanchnic dysoxia during states of shock [13]. The significance of this monitoring for the critically ill was revealed by the landmark study of Guteriez and coworkers, who found that septic shock patients whose gastric CO_2 _did not normalize following resuscitation had a higher chance of dying than did those whose gastric CO_2 _normalized [14]. The physiological mechanisms underlying tissue CO_2 _production was controversial at that time, however, with one school of thought indicating that its origin was due to mitochondrial dysfunction and another school supporting the idea that abnormal CO_2 _reflected an abnormal perfusion of the tissues [15]. However, a number of experimental studies performed by Dubin and coworkers [16] as well as clinical investigations by Creteur and colleagues [17] have now firmly established that elevated tissue CO_2 _reflects a perfusion deficit in the microcirculation. The importance of monitoring the peripheral circulation in the context of distributive shock was further expanded upon by the work of Lima and Bakker, who investigated the clinical significance of assessing peripheral perfusion by physical examination. In so doing, Lima and colleagues identified abnormalities in peripheral perfusion as being associated with high Sequential Organ Failure Assessment scores [18].

## A closer look at the microcirculation

Clinical monitoring of the microcirculation has previously been limited to indirect measures such as lactate, tissue CO_2 _and subjective assessment of peripheral perfusion. Hand-held intravital microscopes offer a different approach [19,20], which incorporates specialized optics such as crossed polarized green light and/or dark-field illumination to filter out the surface reflections as developed much earlier by Slaaf and colleagues and Sherman and colleagues [21,22]. This technology allows for observation of the flowing red blood cells in the microcirculation of the mucosal surfaces of organ beds [19,23]. These hand-held intravital microscopes were subsequently used to directly observe the microcirculation on organ surfaces at the bedside in various clinical scenarios [24-33]. Sublingual microcirculatory observations identified microcirculatory obstructions to be characteristic of septic patients who are resistant to therapy despite corrected systemic hemodynamics [25,32,33]. De Backer and coworkers first demonstrated a correlation between the severity of microcirculatory alterations and morbidity and outcome in septic patients, whereas no such relationship existed for conventional systemic hemodynamic variables [26]. We recently further demonstrated this phenomenon in septic pediatric patients [34]. These findings were reproduced using an early goal-directed therapy treatment by Tryziack and coworkers, who found that an early effective recruitment of the microcirculation predicted Sequential Organ Failure Assessment improvement 24 hours following early goal-directed therapy [25].

Based on the idea that active recruitment of the microcirculation is needed for resuscitation, vasodilatory therapy (for example, nitroglycerin) was shown to be especially effective in recruiting obstructed sublingual microcirculation in pressure-resuscitated septic patients [24]. Similar improvement was not found in fluid-optimized septic patients [35]. In septic shock patients, levosimendan was demonstrated superior to dobutamine for recruiting microcirculation [36], while the addition to norepinephrine of continuously infused low-dose of terlipressin or vasopressin did not affect sublingual microcirculatory blood flow [37].

In cardiac surgery patients, blood transfusions are effective in improving microcirculatory oxygen availability by recruiting previously unfilled microcirculatory capillaries, thereby reducing the diffusion distances between capillaries and tissue cells; this result emphasizes the importance of viscosity in recruiting the microcirculation during resuscitation [38]. The importance of viscosity was further demonstrated in a study of septic patients by Dubin and colleagues, who showed that highly viscous starch solutions can recruit the micro-circulation more effectively than less viscous crystalloids [39]. These studies highlight the unique ability of microcirculatory monitoring to measure not only flow (convection) but also the diffusive capacity of the circulation to transport oxygen by measuring the functional capillary density [31,39,40].

In studies of septic patients, fluid responsiveness has been evaluated at the level of the microcirculation. The type and timing of fluid administration have been found to be an important aspect of fluid efficacy in recruiting microcirculation [39,41]. Vasopressor therapy, although effective in increasing blood pressure, can have limited or even deleterious effects on improving perfusion of the microcirculation [30,42]. One consistent finding from various investigators has been that microcirculatory alterations often manifest themselves at the capillary level by normalized or even elevated flow in the larger venules [24,26,33]. These observations describe the nature of the distributive defect that occurs during shock (especially during the resuscitation phase, as obstructions in the capillary vessels affect the persistence of flow in the larger microvessels) and, furthermore, directly illustrate the nature of the functional shunting that is associated with sepsis and other forms of distributive shock [8]. In particular, the heterogeneity of capillary function has been found by many to be a key characteristic feature of this type of distributive shock [33]. This observation led Tryziack and co-workers to analyze microcirculation images to develop a heterogeneity index to quantify this type of microcirculatory alteration [25].

An additional level of heterogeneity can be attributed to the physiological diversity of the patients themselves. In particular, differences in age influence the response of the patients, with each age group having its own characteristic phenotype and response to critical illness. In this respect, critically ill pediatric and neonatal patients form a special group because they present a completely separate level of (patho)physiological diversity relative to adult patients [43]. For example, as an infant grows during the first years of life, systolic and diastolic pressures are low and heart rates are high. The cardiac output and stroke volume continue to rise until the age of 5 years. Changing cardiovascular physiology is also reflected in the development of the microcirculation, which exists during the initial days and months following birth as a rich network of microcirculatory capillaries that diminishes in density as the infant grows [44].

The response to critical illness is also largely divergent between pediatric and adult patients. A diminished systemic vascular resistance is a hallmark of adult sepsis but is not observed in the pediatric patient. Furthermore, septic shock in pediatric patients, in contrast to adult patients, is often characterized by a hypodynamic response with low cardiac output and high systemic vascular resistance, although a rapid switch can be made. The septic pediatric patient also has a diminished contractile reserve and a poor response to volume loading and inotropic support [43]. Hemodynamic monitoring in these very small patients is indeed a challenging task because the possibilities for invasive hemodynamic monitoring are limited. For instance, Swan-Ganz monitoring has never become common practice in the pediatric age group. Hemodynamic monitoring using hand-held intravital microscopes could offer advantages in these patients; besides targeting an important physiological compartment, this method offers the additional advantage of being largely non-invasive.

The potential application of monitoring the microcirculation in pediatric patients using hand-held intravital microscopic techniques has been exemplified in the work of Top and Tibboel. Using orthogonal polarization spectral imaging, Top and colleagues demonstrated in septic children that persistent microcirculatory alterations were the single most sensitive and specific indicator of outcome [34]. In a recent study, Paize and coworkers further supported the importance of monitoring microcirculatory alterations in pediatric patients by observing that certain microcirculatory alterations in patients with severe meningococcal disease are associated with clinical recovery [45]. Others have shown that some therapies, such as hypothermia and blood transfusions, positively impact the microcirculation of critically ill pediatric and neonatal patients [46,47].

The cardiovascular response of neonatal patients is a largely unexplored area and presents a further level of complexity, not only owing to their small size but also owing to their complex response to hypoxia [48]. This issue is truly a physiological challenge. Whereas hypoxemia is considered a pathological condition in older patients, hypoxia may be viewed as a physiological condition for the neonate, to which the neonate is continuously adapting. Only when these adaptive mechanisms fail does the neonate present as critically ill. These adaptive mechanisms require support that is essential for promoting development [49]. Monitoring the success of the microcirculation at providing blood flow in combination with an assessment of tissue oxygenation is anticipated to form an important platform to realize this support.

## Cell function monitoring

The microcirculation is an integrative physiological compartment in which red blood cells, leucocytes, blood constituents, endothelial cells, smooth muscle cells, parenchymal cells and the intracellular components of these cellular systems integratively and symbiotically function together to ensure optimal oxygen and nutrient transport for the utilization of the parenchymal cells. Indeed, adequate function in terms of perfusion and oxygen transport can be regarded as an indication of success for all of these cellular systems. Microcirculatory dysfunction of this system caused by pathogenic factors, such as inflammation, oxidative stress and hypoxemia, however, can lead to organ dysfunction [12]. Fully understanding the nature of the insult and the indication for appropriate therapy requires insight into the function of the individual subcellular building blocks of the microcirculation. The future of monitoring will need to integrate the functional state of the various cellular constituents into microcirculatory monitoring.

The ability of red blood cells to carry hemoglobin-bound oxygen to the microcirculation is, of course, one of the main functions of the cardiovascular system. The oxygenation state of hemoglobin in red blood cells can be measured quite effectively at the bedside using spectrophotometry, and we used this method to demonstrate the efficacy of blood cell transfusion to improve oxygen availability in the microcirculation in adult anemic hematological patients [50]. Leukocytes form an important source of pathogenic activation, resulting in tissue damage that contributes to organ dysfunction. The ability to monitor leukocyte activation at the bedside using direct observation of their rolling and sticking to the endothelium could therefore provide an important indication of the state of inflammation and possibly the response to therapy. Hand-held intravital microscopy of the sublingual bed was first used for this purpose by Baur and coworkers in patients following the release of the clamp after cardiac surgery [51].

The endothelial cell forms the central regulatory player in the orchestration of the physiology of microcirculation. The cell plays an important signaling role in the regulation of vessel tone in addition to inflammation and hemostasis. Assessment of its function can be accomplished by administering compounds that target endothelial cell function and observing the microcirculatory response. De Backer and coworkers used this approach by topically administering acetylcholine sublingually in septic patients who demonstrated enhanced microcirculatory perfusion [52].

A critical subcellular component that has come to prominence recently due to its relevance to critical illness is the endothelial glycocalyx [53]. This gel-like layer lining the endothelial cells forms the barrier between the intravascular lumen and the endothelial cells. Shedding and disruption of the glycocalyx has been associated with many states of endothelial dysfunction, including the loss of autoregulation and the development of tissue edema and organ dysfunction. Vink and coworkers developed a method to measure the integrity of the glycocalyx by analyzing images obtained from sublingual intravital microscopy [54]. They further developed a software platform to assess the functional state of the glycocalyx directly at the bedside [55].

The routine clinical application of such measurements using the current orthogonal polarization spectral/sidestream dark-field hand-held intravital microscopes and analog video cameras [19,23] has been criticized [56,57]. This criticism is based on the fact that these devices have poor reproducibility and image quality [58,59] and require time-consuming off-line analysis of the acquired images. These devices also suffer from pressure artifacts imposed by the weight of the devices [56,57] and from an inability to implement automatic analysis software to process the generated images [60]. In addition, higher resolution optics and image sensors are required to allow for software analysis and the identification of the subcellular structures associated with microcirculatory function. For these devices to enter the clinical arena, technological advances are therefore mandatory [56,57].

A new hand-held intravital microscope has been developed recently that is based on incident dark-field imaging [22], containing a computer-controlled high-resolution imaging sensor [61]. Such technological advances might possibly address the earlier critiques of the conventional devices but will need to be validated with regard to these critiques before such hand-held vital microscopes can truly enter the clinical arena.

## Towards an integrated physiological monitoring system

The above summary has highlighted the need to extend monitoring of the physiological determinants of organ function from the macro to the micro and down to the cellular level. However, a crucial component of this monitoring is the need to include functional indicators of organ function because it is the successful restoration of organ function that determines the success of intensive care. These indicators of organ function need to be continuous, specific and quantitative. These initiatives are important because they describe a road map for the new developments that are needed to provide complete physiological monitoring of critically ill patients. The information from new sensors and physiological variables, as well as measures of organ function, will require a much higher level of integration than is currently available, and mathematical models of physiology and pathophysiology are expected to play an important role in this integration. From this perspective, these innovations represent a challenge for industry.

By integrating information on all of the characteristics of the patient - including disease, co-morbidities and age - into the evolution of this integrated physiological monitoring system, we anticipate the development of an environment in which the complete continuum of human development, as well as diseases and their response to therapy, can be monitored. Intensive care medicine offers a unique environment for this development, which ultimately may be relevant to other areas of medicine.

## Abbreviations

CO_2_: carbon dioxide.

## Competing interests

CI is the inventor of sidestream dark-field technology and holds shares in MicroVision Medical. He has served as a consultant for this company in the past but has ended all contact for more than 4 years. CI has no other competing interests in this field beyond his commitment to promoting the importance of the microcirculation with regard to patient care. The remaining authors declare that they have no competing interests.

## Declarations

This article has been published as part of *Critical Care *Volume 17 Suppl 1, 2013: Future of Critical Care Medicine. The supplement was proposed by Fresenius Kabi based on presentations from the 'Future of critical care medicine (FCCM) 2012: Today's practice and a look to the future' symposium. Articles were commissioned by the journal, were independently prepared by the authors and have been peer reviewed by the journal. The supplement was edited by John Marini, who declares that he has no competing interests. Publication of the supplement was supported by Fresenius Kabi.
